# Pseudomonas keratitis complicated with spontaneous expulsive suprachoroidal hemorrhage: A case report

**DOI:** 10.1097/MD.0000000000028825

**Published:** 2022-02-11

**Authors:** Ju-Wen Yang

**Affiliations:** a Department of Ophthalmology, Chang Gung Memorial Hospital, Keelung, Taiwan; b College of Medicine, Chang Gung University, Kwei-shan, Taoyuan, Taiwan.

**Keywords:** aged, corneal ulcer, glaucoma, hypertension, suprachoroidal hemorrhage

## Abstract

**Introduction:**

Spontaneous expulsive suprachoroidal hemorrhage (SESCH) is a rare condition. The correlation between SESCH and chronic glaucoma has been reported previously. However, few reports have indicated a correlation between infective keratitis and SESCHs.

**Patient concerns:**

Here, we report the case of an 82-year-old woman with a corneal ulcer who presented with left eye pain for 6 days.

**Diagnosis:**

We found that she has Pseudomonas keratitis and history of chronic glaucoma.

**Interventions and Outcomes:**

During admission, her left eye showed elevated intraocular pressure (IOP). Three days later, the eyeball began to bleed and became painful. She had high blood pressure on that day. Hours after complaints of eye pain, intraocular tissue exposure related to eyeball rupture, and SESCH. The patient underwent evisceration and insertion of a silicone ball for the socket reconstruction. Histopathological evaluation revealed acute inflammation of the cornea and the choroidal vessels.

**Conclusion:**

In elderly patients with infective keratitis and a history of glaucoma and hypertension, it is important to control intraocular pressure and blood pressure and pay attention to the risk of spontaneous expulsive suprachoroidal hemorrhage.

## 1 Introduction

Spontaneous expulsive suprachoroidal hemorrhage (SESCH) is a serious condition. Expulsion of the intraocular contents can cause blindness.

Risk factors for SESCH include age > 60 years, arteriosclerosis, vascular disease, glaucoma, thinning of the anterior eye wall, and intraocular malignancy.^[1,2]^ Previously, SESCH was thought to be caused by both corneal pathology and glaucoma. The pathophysiology of spontaneous expulsive hemorrhage is not well defined. Some authors believe that the bleeding mechanism is related to the rapid and strong displacement of the retina and choroid.^[3]^ Pietruschka and Schill believe that it is due to hemorrhage in the posterior ciliary arteries, leading to increased intraocular pressure (IOP).^[4]^ Pe’er et al believe that it is related to acute necrosis of the wall of the choroidal vessels causing bleeding.^[5]^

Reviewing past reports, we noticed a correlation between Pseudomonas keratitis and SESCH.^[6,7]^ We report the clinicopathological findings and treatment of this patient.

## 2 Case presentation

**Figure 1:**
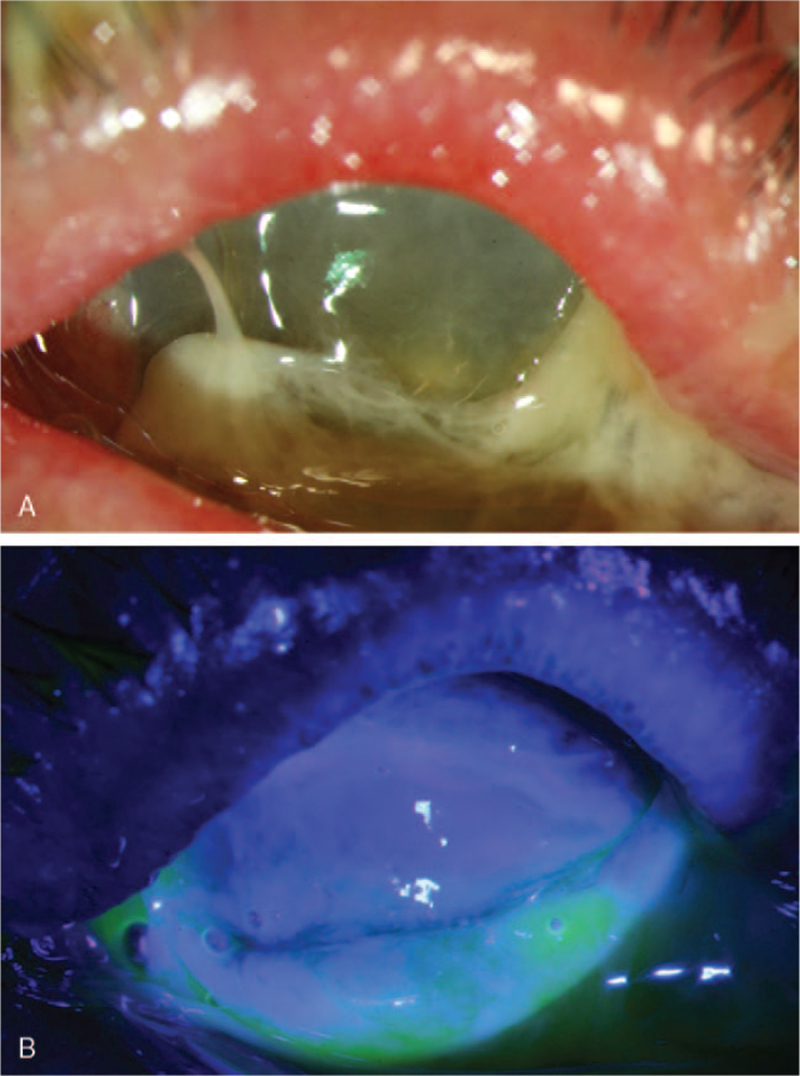
(A) External eye photography of the left eye. (B) Large corneal epithelial defect staining with fluorescein.

**Figure 2:**
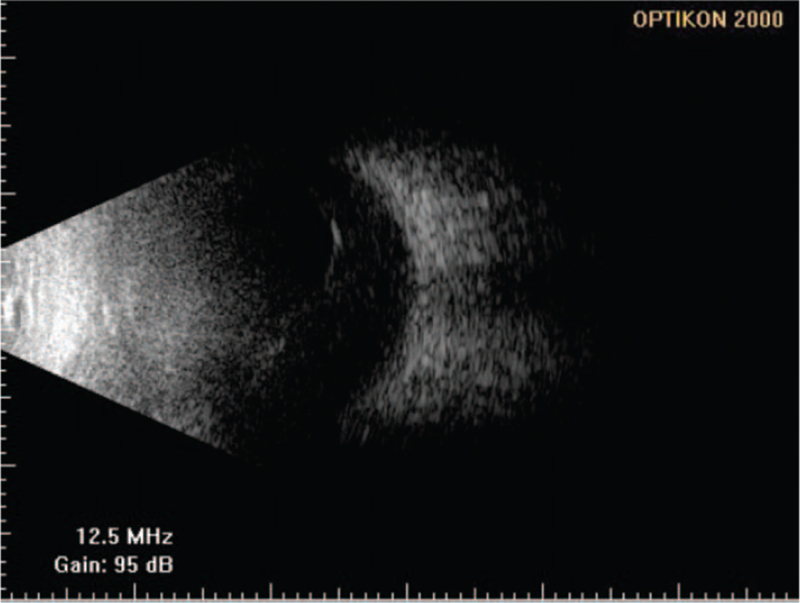
B-scan ultrasonography of the left eye. Mild vitreous opacity.

An 82-year-old Asian woman had infective keratitis in the left eye associated with Pseudomonas aureus infection and a total epithelial defect (Figs. 1 and 2). The patient was then hospitalized for keratitis. The patient had diabetes, hypertension, a left adrenal tumor, hyperlipidemia, and poorly controlled glaucoma. Blindness of the left eye for 5 years has been mentioned for 5 years. Her glycohemoglobin (HbA1c) level was 6.8% 2 months before admission. She complained of pain in her left eye for 6 days. On the first day of admission, intraocular pressure in the left eye was 84 mm Hg. The patient's fellow eye pressure was 24 mm Hg. She received brimonidine eye drops twice daily, bilaterally. Her blood pressure on admission was 173/83 mm Hg, and oral diltiazem 30 mg for blood pressure control was administered in the morning and evening. Other medications included lansoprazole, pitavastatin (2 mg), and metformin (500 mg), administered once daily.

**Figure 3:**
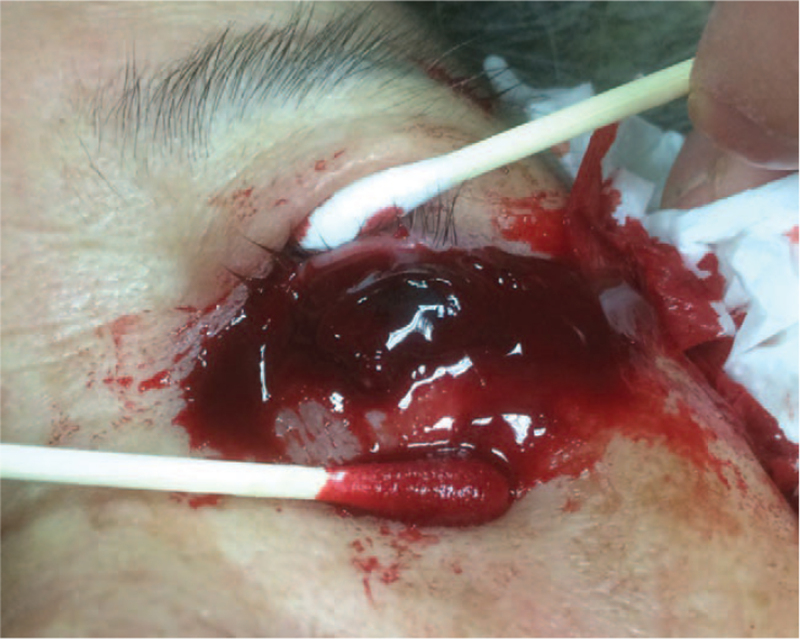
Expulsive suprachoroidal hemorrhage, on day 3 of admission.

Three days after admission, the patient complained of bleeding and pain in the left eye (Fig. 3). No blunt eye injury or force was observed. Her blood pressure was 191/88 mm Hg. Three hours later, the eyeball ruptured and SESCH caused the intraocular content to be exposed. The ruptured wound was located in approximately two-thirds of the limbal region. The patient was administered topical tobramycin eye ointment twice daily for the eye wound and moxifloxacin 400 mg intravenous injection once per day. Three days later, the patient underwent evisceration and insertion of a silicone ball (14 Fr) for socket reconstruction. The ring conformer was placed on the left eye after surgery using tobramycin eye ointment twice per day and pressure patching care for the left eye.

**Figure 4:**
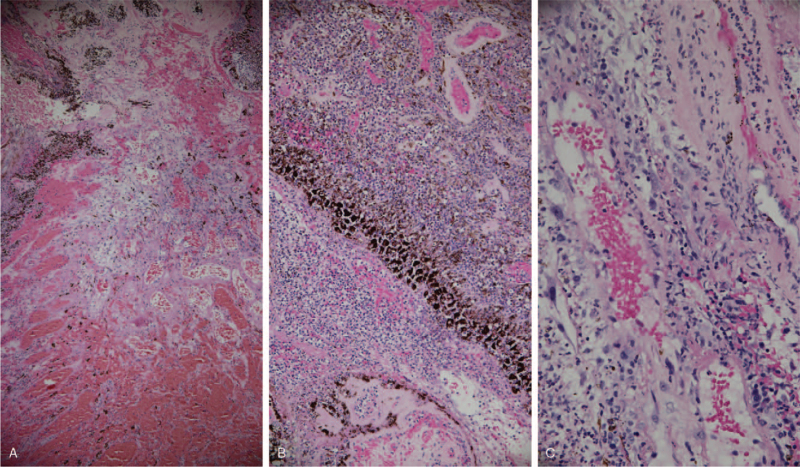
(A) Vascular congestion and hemorrhage of the choroid tissue. (B) Dense acute inflammatory cell infiltration and vascular congestion of the choroid tissue. (C) Acute inflammatory cell infiltration of the vascular wall.

Two days after the operation, the wound was stable and the patient was discharged. She continued antibiotic treatment with oral moxifloxacin 400 mg once daily and tobramycin eye ointment twice daily. Histopathological evaluation revealed acute inflammation of the cornea and choroidal blood vessels (Fig. 4). The patient was followed-up in the outpatient department after discharge. One week after the operation, it was found that there was some yellow discharge in the left eye, we added levofloxacin solution four times per day. The wound discharge gradually decreased. One month after the surgery, the wound was slightly disrupted. The ring conformer was then immediately removed. Two weeks later, the wound healed spontaneously and the ring conformer was reinserted. Three months later, an ocular prosthesis was installed and the appearance was good.

The patient was followed up in the outpatient department for 5 years. Glaucoma in the right eye progressed to end-stage, with optic atrophy and severe visual field defect as the central island (mean defect, −25 dB). Visual acuity was 0.1 and intraocular pressure was 17 mm Hg under medical control with Duotrav at bedtime once per day. The last blood pressure on the return visit was mildly elevated at 158/67 mm Hg. The patient underwent regular medical follow-up.

## 3 Discussion

We encountered a case of Pseudomonas keratitis complicated with SESCH. Histopathological examination revealed acute inflammatory cell infiltration of the vascular walls and vascular congestion in the choroid tissue. Choroidal bleeding, secondary to vascular inflammatory necrosis related to infection, causes SESCH. The intraocular pressure was very high during admission owing to continuous bleeding, which resulted in a large tear in the peripherally inflamed cornea.

In animal models, multifocal choroiditis with retinal detachment consistently occurs after carotid injection of certain bacteria.^[8]^ Pseudomonas aeruginosa can induce moderate to severe fundus lesions after carotid injection. Choroidal capillaries run perpendicularly through the rigid tapetum and then branch at nearly right angles into the choriocapillaris. This area is predisposed to rapid slowing and turbulence of blood flow, and consequently embolization.^[9]^ Large doses of systemic antibiotics failed to prevent lesions in this animal model.^[8]^

According to the clinical manifestations in our patient, vitreous opacity reflected internal bleeding, which led to an increase in IOP. Subsequently, the high blood pressure worsened the overall condition, and the eyeball ruptured. The mechanism of SESCH in this patient is similar to that reported by Pietruschka, Schill, and Pe’er.^[4,5]^

Corneal keratopathy is a devastating risk factor for SESCH.^[1]^. There are few reports of SESCH related to Pseudomonas keratitis. In 2004, Saleh et al reported an unusual case of corneal perforation with crystalline lens extrusion secondary to Pseudomonas keratitis.^[10]^ In 2019, Ting et al reported spontaneous expulsive suprachoroidal hemorrhage in an asymptomatic elderly patient who was confirmed associated with Pseudomonas infection.^[6]^ In 2021, spontaneous suprachoroidal hemorrhage in a patient with pseudomonas keratitis was reported by A Manton et al.^[7]^ All of them became blind once suffered from SESCH.

Pseudomonas aeruginosa is a ubiquitous pathogen that can infect virtually all tissues. A large variety of virulence factors contributes to their importance in eye infections. Prominent factors include pili, flagella, lipopolysaccharide, proteases, quorum sensing, exotoxin A, and exoenzymes secreted by the type III secretion system.^[11]^ Especially the bacterial proteases, streptokinases, and cytolytic toxins are related to the tissue hemorrhage.^[12]^ The association between Pseudomonas keratitis and SESCH deserves further study.

Glaucoma is a known risk factor for SESCH.^[1,2]^ Long-standing glaucoma is associated with abnormal choroidal vessels, including decreased density of capillaries in the choriocapillaris, decreased density of large choroidal vessels, and prolonged blood flow in the peripapillary choriocapillaris.^[13,14]^ The bacterial species on the surface of the eye will also change due to the long-term use of glaucoma drops. It has been reported that the long-term use of prostaglandin analogs for glaucoma may affect the resistance of indigenous flora on the conjunctiva.^[15]^ it may be important to periodically monitor the indigenous flora in glaucomatous eyes treated with eye drops.

Patients with chronic glaucoma infected with Pseudomonas aeruginosa, a highly destructive bacterium, have a high visual threat. The patient was blinded to the occurrence of the SESCH. Early intensive topical antibiotic therapy with fluoroquinolones or fortified antibiotics, including aminoglycosides (e.g., tobramycin), cephalosporins (e.g., ceftazidime), and synthetic penicillins (e.g., carbenicillin), must be considered. Topical antibiotics can stabilize the growth of stromal infiltrates and arrest further stromal necrosis and thinning within 24 to 48 hours.^[16]^

Hypertension is also a risk factor for SESCH.^[1,2]^ Blood pressure control is important. In addition to examining corneal lesions, measurements of intraocular pressure and blood pressure are also important in caring for patients with Pseudomonas keratitis.

## 4 Conclusion

Patients with SESCH have a very poor visual prognosis. To prevent SESCH, regular analysis of ocular surface bacteria in patients with chronic glaucoma and early topical antibiotics used in keratitis patients are suggested. For elderly patients with infective keratitis, attention to hypertension and high IOP is warranted.

## Acknowledgments

We thank Professor Chi-Chin Sun, Ophthalmology Department of Keelung Chang Gung Memorial Hospital for taking care of this patient, and the Pathology Department of Keelung Chang Gung Memorial Hospital for scientific support.

## Author contributions

**Conceptualization:** Ju-Wen Yang.

**Data curation:** Ju-Wen Yang.

**Investigation:** Ju-Wen Yang.

**Writing – original draft:** Ju-Wen Yang.
